# Groundless Fears

**Published:** 1876-03

**Authors:** E. H. Gibbs


### *GROUNDLESS FEARS.*

BY E. H. GIBBS, A.M., M.D.

We are receiving letters from all sections of the country asking medical advice about diseases that exist only in imagination. Two correspondents from Chicago, one from Richmond, Va., and one from Harrisburg, Pa., came to consult us in one week. Three of these persons are as sound and well as it is possible for mortals to be.

We had given them the strongest assurances possible by letter that they " needed not a physician if they had stated their cases fairly." But as an évidence of the ignorance so prevalent in relation to the signs of health and disease, they were alarmed at certain manifestations the absence of which would be evidence of disease.

The truth is, there is a set of unprincipled quacks and rascals who are flooding the country with indecent books and circulars, descriptive of the horrors induced by *evil habits*. They understand that a person who is thoroughly alarmed is easily robbed; and plunder is their object. The plot is well laid. It does not reach the lower stratum of society, where there is but little money and less fear—because greater ignorance—of the retributions of evil doing. But it has produced a world of mental suffering and anxiety among the more intelligent and sensitive classes of both sexes.

The effect of the class of *literature* above referred to is only pernicious. It does evil in ten cases where good results in one. Every physician knows that there are cases, sad indeed, but that the majority suffer far more in mind than in body. Reforms will come in time; and in our opinion the one most imperatively demanded is a censorship over such publications, and the certain and severe punishment of the ignorant villains who thus outrage society. It is said to be the duty of parents to advise and instruct their children, and to warn them against evil. But what son relishes that compound of advise and reproof that the "governor" has dosed him with from childhood. It is far better that advice in relation to the preservation of the health should come from the family physician. If an educated man, he will advise judiciously and treat more skillfully than the quack whose reputation is made by a free use of printer's ink. But the probabilities are four, perhaps ten, to one that the trouble exists mainly in the imagination, and that strict attention to the rules of health will be sufficient treatment. When the general health is good, there can hardly be any serious trouble.

Let any person, not a physician, begin to study his own system and to watch the operations of nature, and he will soon require no other occupation; for he will, in imagination, have all the diseases described in the books, and invent many others that the "faculty" never heard of.

It is folly to ignore the existence of your local physician, if he be a competent one, and send your money to some quack who advertises himself as the "Great Samaritan" or something else equally absurd. If "distance lends enchantment to the view," if your medical "prophet has no honor in his own country," then seek the advice of some one out of the many thousands who are known to be educated and experienced.

